# Promoting Diversity, Equity, Inclusion, and Justice in Grantmaking for Health Care Research: A Pragmatic Review and Framework

**DOI:** 10.1089/heq.2023.0263

**Published:** 2024-06-27

**Authors:** Zoe King, Cati Brown-Johnson, Alica Forneret, Daniel Yang, Elizabeth Malcolm, Daren R. Ginete, Eunice Mercado-Lara, Donna M. Zulman

**Affiliations:** ^1^ Division of Primary Care and Population Health, Department of Medicine, Stanford University School of Medicine, Palo Alto, California, USA.; ^2^ School of Population Health, University of New South Wales, Sydney, Australia.; ^3^ PAUSE, Los Angeles, California, USA.; ^4^ Gordon and Betty Moore Foundation, Palo Alto, California, USA.; ^5^ Kaiser Permanente, Oakland, California, USA.; ^6^ Division of General Internal Medicine, Department of Medicine, Duke University School of Medicine, Durham, North Carolina, USA.; ^7^ Science Philanthropy Alliance, New York, New York, USA.; ^8^ Open Research Community Accelerator (ORCA), San Francisco, California, USA.; ^9^ Haas School of Business, University of California, Berkeley, California, USA.

**Keywords:** diversity equity inclusion justice, research funding, health services research

## Abstract

Funders of research have an opportunity to advance health equity and social justice by incorporating principles of diversity, equity, inclusion, and justice (DEIJ) in their approach to grantmaking. We conducted a pragmatic review to identify opportunities for grantmakers in the health care sector to integrate DEIJ in their funding activities. The resulting framework discusses recommendations within three phases as follows: (1) Organizational Context (i.e., initiate DEIJ efforts within the grantmaking organization, invest in community partnerships, and establish DEIJ goals), (2) Grantmaking Process (i.e., DEIJ-specific practices related to grant design, application, proposal review processes, and support for grantees), and (3) Assessment of Process and Outcomes (i.e., measurement, evaluation, and dissemination to maximize impact of DEIJ efforts). Throughout all grantmaking phases, it is critical to partner with and engage individuals and communities that have been historically marginalized in health care and research. In this article, we describe how adoption of framework practices can leverage grantmaking to advance DEIJ for communities, researchers, and projects.

## Introduction

Despite efforts to address health disparities, the fields of medicine and health services research continue to perpetuate historical conventions that disproportionately disadvantage historically marginalized groups. Meanwhile, pervasive disparities, ranging from delayed kidney transplants for Black patients to access barriers affecting many transgender and gender nonconforming patients,^1–3^ call for research that identifies contributing factors and generates innovative solutions.

Funders of health care research, who hold significant power over the direction of research and the career advancement of researchers, have inadvertently contributed to these disparities by disproportionately underfunding researchers of color^4,5^ and failing to integrate community voices.^6^ Patterns of philanthropic grantmaking resulted in only 7–8% of foundation funding going to people of color and only 1% to Black investigators.^6,7^ By embracing practices that advance diversity, equity, inclusion, and justice (DEIJ) in grantmaking, funders have an opportunity to shape the trajectory of research to advance health equity.

Promoting the principles of DEIJ in grantmaking provides one pathway to advance health equity through research, with potential for far-reaching effects on population health.^8^ There are a number of ways in which applying tailored definitions of these concepts in the context of grantmaking can promote high-impact research. For example, with respect to *diversity*, having a diverse research team that represents a variety of social identities^9^ is associated with higher levels of innovation, a greater focus on health equity, and increased citations of the ultimate research product.^10–12^ Similarly, attention to *equity* throughout grantmaking can help ensure that researchers from different backgrounds have access to resources and opportunities and that community members contribute to research as equitable partners, in turn advancing scholarship focused on health equity.^13^ *Inclusion* facilitates authentic involvement of communities and historically marginalized groups in the research process.^9^ Ensuring inclusive workforces results in improved access to care, patient satisfaction, and outcomes.^14,15^

There are a growing number of articles describing the importance of attending to *diversity*, *equity*, and *inclusion* in grantmaking,^16–19^ but fewer address principles of *justice*. *Justice* focuses on dismantling systems of oppression and rebuilding new systems of equity.^20^ This removal of barriers to resources and opportunities may allow for all individuals to have access to grantmaking opportunities.^21^ The shift from *diversity and inclusion* to a focus on *justice* seeks to create more concrete opportunities for community members and researchers. While often used interchangeably with *equity*, in the context of grantmaking, *justice* entails moving beyond operating under the current circumstances to instead focus on “transformation of circumstances,” for example, through an emphasis on root causes of discrimination, historic harm, and potential solutions.^22^

In order to advance discussions about DEIJ in grantmaking within the health care sector, we conducted a pragmatic review to identify approaches through which funding organizations can integrate DEIJ into funding policy and procedures. Findings are presented in a novel framework that illustrates how DEIJ principles can be incorporated into health care research grantmaking in order to advance health equity. This framework can be leveraged by philanthropic organizations and alliances, academic internal grant programs, federal funding agencies, and other grantmaking institutions that wish to integrate DEIJ into their procedures and track their progress and the outcomes of their efforts.

## Methods

We conducted a pragmatic review, which leverages and adapts systematic review methods to account for more limited resources.^23^ For the review, we searched PubMed in October 2022 for articles discussing DEIJ in grantmaking for health care research.^24^ In consultation with a librarian from the Stanford University Library, we searched for articles written in English from the previous 20 years that focus on DEIJ in the context of grantmaking (search string in Supplementary Appendix S1). One author (Z.K.) screened the articles through three rounds (title, abstract, and full-text screening) to determine if articles met inclusion/exclusion criteria. Inclusion criteria included articles in English, published from 2002 onwards, and discussion of at least one aspect of DEIJ in grantmaking for health care research (full criteria in Supplementary Appendix S2). We also screened the references of accepted articles to identify missing literature (including non-peer-reviewed, gray literature reports).

During the data extraction phase, one reviewer (Z.K.) inductively identified themes in included articles (e.g., funder self-assessment, partnerships, application, measurement, and so on) until thematic saturation was reached.^25^ The reviewer then extracted all relevant data across the identified themes in a data extraction table. All inductive themes were supported by at least three articles. In weekly meetings, three authors (Z.K., C.B.J., D.M.Z.) discussed identified themes, condensing themes where appropriate (e.g., “review” and “selection” inductive themes were combined due to data overlap and grantmaking timeline). Through iterative discussions with experts in research and grantmaking (D.Y., D.G., E.M., A.F., E.M.L.), we synthesized the findings into a framework to follow the structure and process of research grantmaking.

As in other pragmatic reviews, we conducted a quality control review phase.^26,27^ A second investigator (C.B.J.) independently verified 60% of included articles (*n* = 16) to check that they met inclusion criteria. In addition, the second investigator reviewed 20% of the data extraction table (*n* = 5 articles).

In concordance with best practices for scholarship focused on DEIJ, we report on aspects of the authorship team’s self-reported identities that may influence this work.^28^ Our team comprises women and men who identify as Asian, Black, Latine, and white. Collectively, our team has experience in implementation science, health services research, community engagement, and biological sciences. Authors also have different institutional affiliations, including academic institutions (Z.K., C.B.J., D.M.Z., E.M.), health care systems (E.M., D.Y., D.M.Z.), a nonprofit community organization (A.F.), and grantmaking organizations and philanthropy alliances (D.Y., D.G., E.M.L.). We acknowledge that our positionality—including clinical, academic, professional, and other leadership roles—has facilitated this project and influenced our approach to this work. We strive to be aware of our biases and to be mindful of our privilege in conducting this project.

## Results

Of the 2,285 articles screened, 26 met criteria for data extraction (including 18 from search terms and 8 from reference screening). Most articles were set in the context of the United States (*n* = 17), with other articles representing Canada (*n* = 2), the United Kingdom (*n* = 1), and international/multicountry contexts (*n* = 6). Article types included commentaries/editorials (*n* = 13), original research (*n* = 9), reviews (*n* = 2), and gray literature reports (*n* = 2). Articles were written by researchers (*n* = 13), representatives from funding organizations (*n* = 6), and a combination of researchers, funders, nonprofit organizations, government agencies, and educational organizations (*n* = 7). Themes from the review were grouped into three phases. The first phase, *Grantmaker’s Organizational Context*, included the following themes: Initiate and Sustain Internal DEIJ Efforts (represented by *n* = 9 articles); Invest in Community Partnerships (*n* = 16); and Establish and Communicate DEIJ Definitions and Goals (*n* = 6). The second phase, *Core Components*, included Grant Design (*n* = 12); Outreach and Application (*n* = 6); Review and Selection (*n* = 8); and Support for Applicants and Grantees (*n* = 6). The third phase, *Assessment of Process and Measures*, included Measure (*n* = 5), Evaluate (*n* = 5), and Disseminate (*n* = 5). We organized these themes into a framework for promoting DEIJ in grantmaking (Table 1). A full list of recommendations for promoting DEIJ through grantmaking is reported in Table 2.

**Table 1.** Framework to Promote DEIJ in Grantmaking for Health Care Research

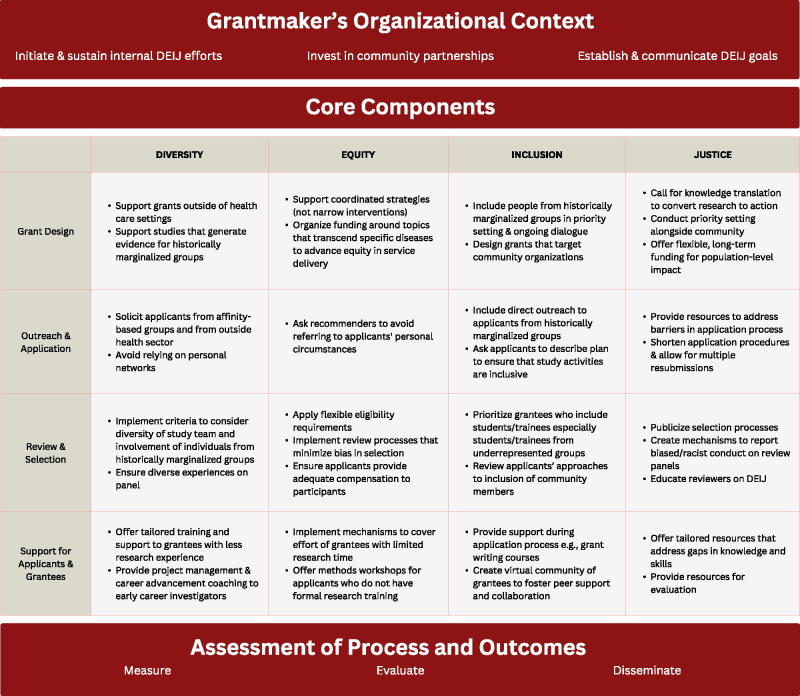

DEIJ, diversity, equity, inclusion, and justice.

Source: Narrative Review, 2022.

**Table 2.** Recommendations to Promote DEIJ in Grantmaking for Health Care Research

ORGANIZATIONAL CONTEXT	
Initiate and sustain internal DEIJ efforts	Examine board and staff diversity^29–30^ and make a concerted effort to ensure equal representation of all racial and ethnic groups across the entire funding agency, not just specific centers or efforts.^31^ Consider engaging in group hires as single minoritized individuals are subject to harm.^6^ ○ “A foundation can be diverse but not culturally competent, but cannot be culturally competent without being diverse”.^30^ Prioritize transparency and accountability.^32^ Engage in cultural humility, involving the following components^6^: ○ Lifelong commitment to self-learning and critical self-reflection ○ Dismantling inherent power imbalances and building respectful relationships between patients and clinicians ○ Developing mutually beneficial nonhierarchical clinical and advocacy partnerships with communities ○ Creating institutional alignment and accountability Engage research participants and individuals who are the focus of study as equal partners in the scientific process.^6^ Continually conduct internal health equity readiness assessments on their own organizational characteristics, workforce competencies, culture, and operating mechanisms that obstruct or advance Black-centered racial justice and reproductive health equity.^6,33^ Engage in an antiracist process evaluation of existing philanthropic policies, practices, and programs that perpetuate the exclusion and erasure of historically marginalized scientists. Determine areas of alignment and disconnect between program design, inputs, activities, and outputs to decolonize power structures and strengthen funder alignment and accountability to the needs and priorities of the most impacted communities.^6^ Develop and publicize a set of ethical principles that guides where funds are allocated to (e.g., public health research vs. health research).^34^ Identify DEIJ champions (including staff, CEO, board members, grantees, partners) and provide resources for champions to deepen partnerships, spread information, write strategic plans, and ask challenging questions.^30^ Implement well-facilitated staff and board conversations and trainings on structural racism and provide opportunities for self-reflection and sharing of personal experiences.^33^ Empower staff and leadership to identify and respond to racism on review panels and elsewhere.^35^ At all levels, funders can be committed to reflect practice about their own social location, power, and privilege and refine their approach accordingly.^36^ Ensure that all staff understand the history of racism in the United States, as well as the cultures of Indigenous territories in which grantmaking takes place.^36^
Invest in community partnerships	Center the wisdom, assets, and leadership of communities that bear the burden of oppression rather than grantmakers without lived experience when deciding what needs to be prioritized and how grants are structured.^6,29,30,32,33,36–44^ Engage communities in a meaningful way during the design, selection, and execution of research activities: ○ Diverse stakeholders may include youth, grassroots organizations, faith leaders, business community, patients, clinicians, and others who are generally not at the table when making decisions.^30,44^ ○ Community members can be involved in all stages of the research process, from topic selection to research design and activities to dissemination.^44^ Community members can have the power to make decisions, guide human/money/time resources, and call for a pause on work when necessary.^6^ ○ Community advisory boards or citizen panels are not automatically partners if they are only reacting to agendas handed to them.^6,44^ ○ Take the time to develop trusted and sustainable partnerships through flexibility, support, relationship development, and shared agenda-setting activities.^32,42,44^ ○ Develop ethical conflict resolution strategies and principles of partnership before beginning any collaborations.^6^ ○ Implement community-based IRBs where possible to examine institutional alignment and accountability to racial and reproductive justice.^6^ ○ Community members can inform payment strategies to participants, including for reimbursement of expenses incurred as a result of participation, compensation for time and efforts, and incentive payments to encourage participation and retention.^39^ ○ Develop processes for gaining authentic and productive feedback.^30^ Create and sustain public–private partnerships, e.g., matching funds for federal dollars and bolstering support for riskier projects that encounter political resistance.^29,33^ Engage with sectors outside of those directly related to health, e.g., housing, education, transportation, community development.^29,30,45^ Encourage collaboration between investigators of different experience levels and racial and ethnic groups for a mutually beneficial opportunity for colearning and mentoring.^31^ Support collaborations between majority and minority institutions and provide mechanisms to support those collaborations.^46^
Establish and communicate DEIJ definitions and goals	Establish a set of core values that demonstrate long-term commitment to DEIJ (e.g., racial justice, flexibility, adaptability, diversity, partnership, celebration of culture) and then develop specific processes for integrating those values into internal procedures, aligning with staff values, and incorporating into external grantmaking.^30^ Developing a theory of change can help grantmakers to operationalize their values and strive for the intended impact. When developing their theory of change, grantmakers must grapple with the question of whether health equity is a process (e.g., core values of equity being reflected in grantmaking procedures), an outcome (e.g., eliminating health inequities), or both.^30^ Shift from a narrow focus on disparities to a clear set of values that put DEIJ at the center and clearly define.^29^ Shift thinking from disparities to equity and justice by funding projects that focus on social and structural determinants of health.^29,30^ Explicitly state racism as a determinant of health.^29^ Define and promote research justice^6^: ○ 1. Values and amplifies cultural and spiritual; mainstream; and experiential knowledge ○ 2. Examines relationships and intersections between research, knowledge construction, and political power ○ 3. Centers community experts as vital partners in knowledge construction and self-determination; community mobilization; and social transformation and policy reform Highlight and advance the ways that research can promote justice^47^: ○ 1. Laws and policies that are informed by high quality research are less likely to treat groups unfairly ○ 2. Health research can motivate policy change by highlighting injustices ○ 3. Can improve people’s lives thereby reducing inequality Funders can publicly signal that they are committed to DEIJ.^40^ In public facing materials, explicitly state that racism persists in the U.S. research field and that the organization is actively working to expel racism.^35^
**CORE COMPONENTS**	
Grant Design	Consider how grant proposals can include calls for knowledge translation to facilitate long-term goals of converting research into action.^47^ Priority setting requires meaningful inclusion of individuals from diverse backgrounds, including community members.^43^ ○ Engage in dialog with faculties at Historically Black Colleges and Universities and other minority serving institutions to better understand their priorities.^46^ Design grants that facilitate long-term change by creating more flexible funding structures, supporting policy change, and developing public–private partnerships.^29^ ○ A multiple-phase approach to funding can facilitate long-term change, e.g., Phase 1 focused on initial design, development, and testing of an intervention; Phase 2 focused on full implementation to additional sites and evaluation; and Phase 3 providing funding for scale-up and sustainability.^42^ Organize funding schemes around different types of research rather than different diseases to avoid fragmentation and promote non-disease specific interventions that are needed to achieve equity.^34^ Build in flexibility to the grant structure to allow grantees to identify and learn from what worked and did not work to establish course corrections.^42^ Allow for grants outside of the health sectors, e.g., education, housing, transportation, criminal legal system, etc. to address upstream factors.^29,33,37,45^ Design grants that are targeted to smaller community organizations, rather than large academic centers.^38^ Enable grants that seek all kinds of evidence, e.g., qualitative and quantitative research, social science research, practitioner knowledge, and traditional/community knowledge.^34,36^ For grants targeting racial and ethnic disparities, interventions that are culturally responsive, support patient navigation, and include a variety of clinicians are more likely to be successful.^29,48^ Highlight the rationale for the grant structure, including rationale for selected issue area, program approach, and project activities to internal and external stakeholders.^36^ Develop calls for research that support multiple coordinated strategies for equity rather than narrow interventions.^33^
Outreach and Application	Broaden the distribution of funding announcements to include direct outreach to affinity-based societies, institutions and applicants from underrepresented groups, journals routinely accessed by historically marginalized investigators, social media, and informational webinars.^16,46^ Develop enhanced communication strategies to engage with nonhealth groups that are also working to advance racial justice.^33^ Avoid relying on personal networks and relationships for outreach.^34^ Convey attributes of successful applicants using nongendered language, e.g., changing “leadership potential” to “promise to make significant contributions”, “importance” to “influence”, “innovation” to “originality”, and “creativity” to “inventiveness.”^49^ Ask that recommenders comment about the applicant’s record avoiding referring to personal circumstances, e.g., marital status, age, work-life balance, and roles outside professional setting.^49^ Ask for diversity or resilience statements^16^: ○ Resilience statements: invite applicants to share their experience, attributes, and competencies to help reviewers contextualize their achievements and/or career trajectories. ○ Diversity statements: require all applicants and potentially department chairs and mentors to explain how they promote DEIJ in their environments. Allow for multiple resubmissions.^16^ Revise reviewer comments to make feedback more constructive for future improvement.^16,46^ When relevant ask applicants to use tools such as the INCLUDE Ethnicity framework for trial teams to think about which ethnic groups to include in their trials in order for results to be widely applicable.^40^ Simplify application procedures as much as possible.^46^ Provide information in the application on the importance of including historically marginalized groups as participants as previous research showed that researchers may perceive inclusion as too difficult, too expensive, or not relevant. Funders can provide resources to overcome barriers to inclusion.^40^
Review and Selection	Include specific criteria focused on planning for inclusion of historically marginalized participants, as well as diversity of the study team (particularly for investigators who represent the communities being served) and diversity of institutions.^6,31^ ○ All forms of diversity of the study team can be considered, e.g., race, ethnicity, gender, sexual orientation, disability, career stage.^35^ ○ Diverse teams can be prioritized for funding and their applications can be automatically slated for discussion by an automatic system or the scientific review officer.^35^ Allow for flexibility around application eligibility requirements to allow for life circumstances that may have affected career trajectories, e.g., not counting periods of medical or parental leave.^16^ Prioritize grantees that include trainees (beginning at high school or undergraduate level) especially those for historically marginalized groups.^16^ Implement review process that minimizes bias in selection through methods such as: ○ Blinded review and anonymized applications^16^ ○ Using second-level review to provide oversight to ensure the funding decision is objective to ensure adequate inclusion of grantees from historically marginalized groups.^31^ ○ Equally distributing funding to all qualified researchers without selection.^50^ ○ Relying on expert administrators to directly select proposals rather than seeking advice from external experts.^50^ ○ Focusing only on applicants’ past performance rather than judging validity of proposed project.^50^ ○ Proactively identifying suitable applicants and asking them to apply for funding.^50^ ○ Implement anti-bias training for reviewers.^16,50^ ○ Publishing clear conflict of interest policies.^50^ ○ Providing reviewers with clear guidelines on evaluation criteria.^50^ ○ Include an ambassador trained on racism in all panels.^35^ Prioritize projects that use budget line items and narratives to reflect allocation of money and resources to community leaders.^6^ Publicize processes for selection to increase transparency.^34^ Eliminate any bias in selection criteria that may prioritize certain kinds of research, e.g., clinical trials over others, e.g., social science research.^34^ Select applicants who are providing adequate compensation to their participants to increase inclusion of historically marginalized groups.^39^ Ensure that diverse experiences and perspectives are represented on the review panel.^16,35,46^ Create efficient mechanisms for reporting racist or biased conduct during and after review panels, including developing a standardized policy to remove reviewers with a history of offenses from reviewer pool, publicizing policies, and effective follow-up.^16,35,46^ Educate reviewers about DEIJ and minority serving institutions, e.g., Historically Black Colleges and Universities.^46^
Support for Applicants and Grantees	Provide opportunities for additional research training of applicants as needed to increase award attainment.^49^ Identify ways to guarantee protected research time to enhance funding outcomes.^49^ Provide resources during the application process such as courses or webinars that highlight best practices in grant writing, including courses targeted for historically marginalized investigators.^16,46^ Enable the provision of appropriate resources as needed, including community engagement expertise, letters of support, pilot project funds, access to biostatistics expertise, and so on.^32^ Provide workshops for funded projects to meet with experts and provide structured lessons on implementation science, partnership development, scale-up, and evaluation.^42^ Provide resources for evaluation, including engaging an evaluation expert in each funded project.^42^ Tailor resources to individual projects rather than providing generic resources and toolkits due to the potential for project diversity across context, population, and intervention.^42^ Provide career coaching to early-stage grantees, e.g., online office hours with a coaching director, grant writing webinars to provide budgetary expertise.^51^ Create a virtual community of grantees to provide social support to early career investigators.^51^
**PROCESS ASSESSMENT**	
Measure	Include comprehensive measurements that assess long-term impact, including intervention evidence and evaluation, reach and scale, organizational capacity, partnership development, system readiness, community context, cost factors, and knowledge development and exchange.^6,42^ Track DEIJ measures over time, e.g., percentage of grants that support health equity, health outcomes of populations, demographics of grantees.^16,29^ ○ When collecting demographic information (gender, race, ethnicity, sexual orientation, SES, disability status) of grantees and study teams, use respectful language, include the option not to respond, and allow for self-identification.^16^ ○ Establish more inclusive demographics categories by seeking input from communities.^16^ ○ Be transparent about how the demographic data will be used (e.g., to track diversity of cohorts, part of the review process).^16^ ○ Collect demographic information of other stakeholders outside of grantees, including organizational leadership (advisory boards), staff, volunteers, and review committees.^16^ Develop measurements for capturing vested partnerships, including agenda setting rather than the number of partners in a network.^42^ Develop measures that align with a theory of change that details context, assumptions, activities, outputs, program theory, and hypothesized mechanisms of change and intended outcomes.^36^ While there are few metrics that measure social justice,^30,31^ grantmakers can consider tracking funding to individuals and groups that have been historically excluded from funding opportunities, such as small community organizations.^38^
Evaluate	Continually monitor effects of grantmaking from a health equity perspective and to allow for continuous learning.^29,36^ Improve tracking of successful and unsuccessful applicants.^46^ Evaluate all DEIJ initiatives to determine if they indeed result in desired outcomes.^16^ Be aware of inadvertent harm that may result from funded interventions and evaluate changes through this lens.^16^ Revising their theory of change may help grantmakers evaluate their organization’s impact.^30^
Disseminate	Publicly share successes and failures to learn from other DEIJ-specific grantmaking efforts.^16^ Build the infrastructure and capacity of groups to engage elected officials and push for policy change.^33^ Disseminate information through a variety of channels outside of peer-reviewed publications, e.g., lay publications, presentations, social media, community organization activities, policy-related articles, curricula, trainings.^52^ Use power as a grantmaking organization to bring together grantees, partners, and community leaders, building new networks and coalitions and promoting advocacy.^29^ There may be opportunities to facilitate and fund publication of innovative, nontraditional research.^41^

DEIJ, diversity, equity, inclusion, and justice.

### Grantmaker’s organizational context

#### Initiate and sustain internal DEIJ efforts

In order to promote DEIJ within funded projects, grantmakers must first look internally at their organization’s DEIJ practices and prioritize transparency and accountability.^33^ Leadership can evaluate their existing philanthropic practices and policies for activities that perpetuate the exclusion of scientists from historically marginalized groups.^6^ Concerted efforts may be needed to ensure representation of multiple racial and ethnic groups across the entire grantmaking organization; efforts should include examining the diversity of board members^29,30,33^ and should not be limited to specific centers or activities.^31^ At all levels, grantmakers can reflect on their own power and privilege and ensure that all staff are informed about key issues influencing health disparities, such as racism and other structural determinants of health, as well as the cultures of Indigenous territories in the regions involved in the grantmaking process.^36^

#### Invest in community partnerships

The most prominent theme among articles was the importance of elevating the wisdom, assets, and leadership of communities that bear the burden of oppression when deciding what needs to be prioritized and how grants are structured.^6,30,32,33,36–44^ This involves eliciting the perspectives of community partners, leveraging their experiences and strengths, and ensuring that their voices are central to decision-making. Community advisory boards and citizen panels (which may be physically co-located or dispersed but joining together on conference calls) can be included as equal partners, and not limited to a role in which they only react to grantmaker or researcher agendas.^6,44^ To operationalize this, community members can be involved in all stages of the research process, from topic selection, to research design and activities, to dissemination.^44^ Grantmakers may also benefit from other forms of partnerships such as public–private partnerships;^29,33^ engagement with sectors outside of health (e.g., housing, education, community development, and so on);^29,30,45^ and collaborations between predominately white institutions and institutions serving historically marginalized groups, e.g., Historically Black Colleges and Universities, that are mindful of power and resource differentials between institutions.^46^

#### Establish and communicate DEIJ definitions and goals

Grantmakers should establish a set of core values that demonstrate long-term commitment to DEIJ (e.g., racial justice, flexibility, partnerships, celebration of culture, and so on).^30^ Once values are established, grantmakers can develop specific processes for integrating those values into internal procedures and incorporating them into external grantmaking efforts.^29–31,33,34^ Developing a theory of change can help grantmakers to operationalize their values and strive for the intended impact.^30^ In addition, when establishing values and goals, organizations should consider where they can shift their priorities from a narrow focus on health disparities to a broader mission of achieving health equity and justice.^29,30^ Finally, grantmakers should publicly signal their commitment to DEIJ, and when relevant to specific priorities and projects, grantmakers should explicitly describe racism as a determinant of health.^29,40^

### Core components

#### Grant design

A variety of people, including community members, should be involved in priority setting to ensure that DEIJ is central to the grant design process.^43^ It can be helpful for grantmakers to engage in dialog with individuals from institutions serving historically marginalized groups to better understand their priorities.^46^ Grants are more likely to facilitate long-term and systems-level change if grantmakers create more flexible funding structures.^29,42^ For example, the Public Health Agency of Canada’s Innovation Strategy implemented flexibility by establishing a multiphase approach as follows: Phase 1 supported the initial design, development, and testing of interventions; Phase 2 supported the implementation, delivery, and evaluation of interventions; and Phase 3 supported the scale up of interventions.^42^ Grant design can also take into account calls for different types of evidence (e.g., qualitative and quantitative research, practitioner knowledge, and community knowledge),^34,36^ as well as directly targeting smaller community organizations to lead projects, rather than just large academic centers.^38^

#### Outreach and application

In order to allocate resources justly and equitably, grantmakers can consider competitive applications and avoid relying on personal networks.^34^ Outreach can be directed to affinity-based societies, institutions serving historically marginalized groups, social media, and informational webinars.^16,46^ Simplifying and shortening application procedures may reduce barriers for applicants of all backgrounds, including researchers and community members.^46^ There are several ways that grantmakers can adapt the application process to incorporate DEIJ principles, for example, by requiring diversity or resilience statements in applications,^16^ allowing for multiple resubmissions,^16^ and conveying attributes of successful applicants using nongendered language.^49^ When relevant, grantmakers can ask applicants to use tools such as the INCLUDE Ethnicity framework for clinical trial teams to think about diversity of participants.^40^

#### Review and selection

To ensure that the review process incorporates DEIJ considerations, grant review instruments can include a field to discuss the degree to which proposals include historically marginalized participants, as well as criteria regarding diversity of the study team and diversity of institutions.^6,31^ Grantmakers should also implement review processes that minimize bias in selection through methods such as blinded review and anonymized applications;^16^ second-level review to provide oversight;^31^ equal distribution of funds to all qualified applicants;^50^ implementing anti-bias training for reviewers;^16,50^ and including an ambassador with expertise in racism or other forms of discrimination in all panels.^35^ Review panels should reflect diverse experiences, identities, and perspectives, and grantmaking organizations should implement a mechanism to report concerns about discriminatory conduct and biased selection patterns such that reviewers who exhibit these behaviors can be removed from panels.^16,35,46^

#### Support for applicants and grantees

There are a number of effective strategies to support applicants with less experience in grant writing, including offering courses that highlight best practices in grant writing, facilitating community engagement, and providing access to biostatistics expertise and proposal development consultation with an experienced researcher.^16,32,46^ One grantmaker found that due to the diversity of projects across population, intervention focus, and context, generic toolkits and resources were not as effective as tailored approaches to resource provision.^42^ Early-stage grantees may want additional coaching and peer support around project management (e.g., budget development, regulatory considerations), community partnership development, and career advancement, which can sometimes be achieved through building a virtual community of grantees.^51^

### Assessment of process and outcomes

#### Measure

In order to ensure that DEIJ-related efforts have the intended impact, it is essential for grantmakers to track measures that relate to their goals, such as the demographics of their grantees, the percentage of their grants that focus on health equity, health outcomes of historically marginalized populations, the number of projects that are able to influence policy change, and the generation of research products outside of academic scholarship that influence community health (e.g., lay publications, testimony, presentations, and so on).^16,29,42,52^ While there are few metrics that measure social justice,^29,30^ grantmakers can consider tracking funding to individuals and groups that have been historically excluded from funding opportunities, such as small community organizations.^38^ Grantmakers can consider collecting demographic data of stakeholders outside of grantees, including community partners, and can continue to promote inclusiveness in demographic categories by seeking community input.^16^

#### Evaluate

Repeated monitoring of the effects of grantmaking from a health equity perspective can allow for continuous learning.^29,36^ All DEIJ initiatives should be evaluated to determine if they result in desired outcomes.^16^ Grantmakers should follow-up with successful and unsuccessful applicants to evaluate the outcomes of their efforts to advance DEIJ.^46^ Grantmakers and grantees should also proactively monitor for inadvertent harm that may result from a funded intervention.^16^ Revising their theory of change may help grantmakers evaluate their organization’s impact.^30^

#### Disseminate

Publicly sharing successes and failures will help grantmakers learn from one another.^16^ Information about grantmakers’ DEIJ journeys, as well as results from funded projects, can be disseminated through a variety of ways outside of peer-reviewed journals (e.g., lay publications, presentations, social media, community organization activities, policy-related articles, and trainings).^52^ There may be opportunities to facilitate and fund the publication of innovative, nontraditional research.^41^ Grantmakers can use their leverage to bring together grantees, partners, and community leaders to build new networks and coalitions to promote advocacy.^29^ Grantmakers can also help to build the capacity for groups to engage elected officials to push for policy change.^33^

## Discussion

In this article, we offer a framework to support efforts to promote DEIJ and advance health equity through grantmaking (Table 1). Our pragmatic review and synthesis yielded specific steps that funders can take to advance DEIJ within their own organizations, through the grantmaking process, and through measurement, evaluation, and dissemination. Many of these recommendations are already being practiced. For example, the Patient Centered Outcomes Research Institute emphasizes the importance of patient voices, and the National Institutes of Health established a UNITE initiative to address structural racism within the institute.^53,54^ Furthermore, non-peer-reviewed reports from grantmaking organizations such as the Open Society Foundations, Robert Wood Johnson Foundation, and the Ford Foundation (not reported herein) echo findings from this review.^18,19,55–60^ The proposed framework offers additional opportunities to promote DEI and to expand current efforts to also address justice throughout the grantmaking process, from internal assessment to dissemination of research incorporating DEIJ principles. Practically, use of this proposed framework could look like a structured review of each stage of the grantmaking process to see where grantmakers are already incorporating some of these practices and where there are opportunities to incorporate new practices. While organizational capacity may vary and funders may need to prioritize certain practices for feasibility reasons, this framework and the accompanying list of practices offers a resource for funders to identify gaps and consider potential targets for intervention.

One of the most foundational themes emerging from our work is the importance of involving community members at all stages of the grantmaking process.^6,30,32,33,36–44^ This engagement must be authentic, meaningful, and appropriately compensated.^39^ Surface-level community involvement can result in tokenism and harm to Black, Indigenous, and other historically marginalized communities.^6,61–63^ Deep partnerships, by contrast, extend beyond the mere presence of a community advisory board, ensuring appropriate elevation of the voices of people impacted by grantmaking products and outcomes.^6,44^ Inclusion of community members is also shown to strengthen the rigor, relevance, and reach of science.^64^ Other practices that may strengthen community partnerships include setting funder values around collaboration before establishing partnerships; establishing processes for how to leave a partnership when it is not working; evaluating how funder values are being received by the grantees and the community; and reviewing language for inclusivity in all sections of the grant application e.g., educational requirements. When funding community organizations, grantmakers should—when possible—offer tangible support for grant management. Developing partnerships with community organizations such that they can serve as grantmakers themselves may also facilitate capacity building.

Notably, the framework we have developed extends beyond diversity, equity, and inclusion and incorporates *justice* as an additional domain. Some of the justice-focused practices (e.g., conducting priority-setting activities alongside community members; shortening application procedures; offering flexible, long-term funding for population-level impact)^29,35,43,46^ serve to remove barriers in an attempt to equalize opportunities for researchers and community members from historically marginalized groups to obtain funding. Practices such as auditing reviewers for signs of bias aim to address potentially discriminatory behavior.^35^ Other practices focus on achieving justice by allocating resources based on individuals’ circumstances, for example, by providing tailored resources that address gaps in knowledge and skills or offering intensive grant management support for those with less research experience.^16,32,42,46^ There may be opportunities for more transformational practices as well, such as providing grant funding directly to community members instead of academic institutions. Over the past four years, many funders have integrated DEI into their grantmaking processes; by expanding beyond DEI to focus on justice, funders have an opportunity to dismantle systems of oppression and address barriers to equity by implementing transformative practices. Future evaluations should examine the relative merit of these efforts and explore potential unanticipated adverse consequences.

This article also extends existing approaches by emphasizing reflection and sustainability. Ultimately, the goal of funding in health care research is to develop solutions—ones that will stand alone without additional grant-funding and provide sustainable change poststudy.^65^ For this framework, we specifically explore measurement, evaluation, and dissemination as levers to promote sustainability. This is a challenging but necessary area for grantmakers who aim to advance DEIJ; indeed, other widely accepted intervention and behavior change frameworks in health care such as the RE-AIM framework in implementation science also struggle with sustainability or “maintenance.”^66,67^ Perhaps a framing of sustainability within continuous learning is less daunting. Most health care organizations are familiar with the concept of Learning Health care Systems, which leverage metrics and qualitative insights for continuous improvement.^68^ Community-based organizations are likely to also incorporate concepts of lifelong learning that can be highlighted in grantmaker-community partnerships. Robust internal evaluations by grantmakers can be time-consuming; thus, hiring external evaluators may help to facilitate this process. Indeed, we contend that without intentional feedback and dissemination, grantmakers risk failed projects with minimal impact.

Finally, while this framework provides concrete recommendations for improving grantmaking processes, it is important to acknowledge how DEIJ efforts have previously failed. In the grantmaking space, grantmakers have disproportionately underfunded researchers of color, particularly Black researchers.^4–7^ In academic institutions, DEIJ efforts were built off the unrecognized labor of faculty of color, particularly women of color.^69^ As recounted by Dr. Angie Beeman, academic departments have engaged in problematic diversity and inclusion efforts by allowing white faculty to take visible leadership roles in DEIJ without recognizing or compensating women of color for the true DEIJ work.^69^ There is also a pattern of institutions asking their faculty of color to lead DEIJ initiatives without adequate time or compensation, a practice that creates what is referred to as a minority tax.^70^ Surface-level, performative activism or “health equity tourism” may be a constant risk;^71,72^ investment in “racism-centered intersectional” approaches that provide structural change may be the best way to ensure true engagement with DEIJ principles.^69^

This work has several limitations. First, we conducted a comprehensive but by no means exhaustive review of the literature. Because we conducted a pragmatic review, which adapts systematic review methods to account for resource limitations, most articles were reviewed by a single reviewer; although our validation phase included a second reviewer for 60% of screened articles and 20% of our extraction table, future systematic reviews on this topic could seek to address this limitation with additional resources. Second, while we defined diversity broadly, many of the included articles centered around race and ethnicity and/or gender. Additional work could seek to address intersectionality and multiple marginalization by including other identities such as LGBTQIA+ individuals and people with disabilities in broader definitions of diversity and inclusion.^73,74^ Relatedly, as this framework originated from a review of the literature—which may include but does not always center community voices and perspectives—it is important that future work on DEIJ in grantmaking is developed by Black, Indigenous, and other people of color, as well as individuals with additional marginalized or intersectional identities. Future work could: 1) include principles of Accessibility and Belonging;^9,75^ 2) integrate recommendations from adjacent fields (e.g., trauma-informed principles^76^ and restorative justice^77^); 3) establish the evidence-base (from community and research perspectives) of these recommendations; 4) adapt this framework to sectors outside of health care; and 5) interview funders themselves to understand existing DEIJ efforts and to identify gaps that might warrant additional recommendations not documented in literature.

In conclusion, applying principles of DEIJ throughout the grantmaking process offers a pathway to advance social justice and health equity at a broader scale—across topics and institutions. This framework provides concrete recommendations for promoting DEIJ, when used authentically and guided by partnership with historically marginalized communities. The recommendations demonstrate numerous ways health care grantmakers can implement DEIJ in their work, allowing funders to choose practices that they think are most aligned and best fit with their current organizational structures and mission. Co-design and community-based participatory research may be used among other tools to ensure that future work centers impacted individuals. We encourage grantmakers to use this framework in conjunction with other models to continually promote health equity and social justice.

## Supplementary Material

Supplementary Appendix S1

Supplementary Appendix S2

## Abbreviations Used

CEO: Chief Executive Officer
DEIJ: Diversity, Equity, Inclusion, and Justice
LGBTQIA+: Lesbian, Gay, Bisexual, Transgender, Queer, Intersex, Asexual, and all other identities not encompassed in the short acronym
