# Supplementary material for: Promoting Diversity, Equity, Inclusion, and Justice in Grantmaking for Health Care Research: A Pragmatic Review and Framework
Source: Health Equity. 2024 Jun 27;8(1):391–405. doi: 10.1089/heq.2023.0263 (PMC11250833; doi:10.1089/heq.2023.0263)
Supplement: Supplementary Appendix S1 [file heq.2023.0263_appendix.docx]

APPENDIX

Appendix Exhibit A1. Review Methodology: Search String

Search string:

(justice[tiab] OR social justice[Mesh] OR equity[tiab] OR inequit*[tiab] OR health equity[mesh] OR diversit*[tiab] OR cultural diversity[mesh] OR inclusive[tiab]) AND (grantmak* OR "grant mak*" OR "grant-mak*" OR funder*)

Appendix Exhibit A2. Review Methodology: Screening Process

| **Screening Round** | **Inclusion Criteria** | **Exclusion Criteria** | **Accepted (A)** | **Uncertain (U)** | **Rejected** | **Total A/U** |
| --- | --- | --- | --- | --- | --- | --- |
| Total Results from Search |  |  | 2258 |  |  | 2285 |
| Title Screening | General focus on DEIJ in grantmaking for healthcare research | Published before 2002; not written in English | 13 | 40 | 2205 | 53 |
| Abstract Screening | Specified focus on aspects of DEIJ in grantmaking for healthcare research | Abstract not in English; lack of focus on healthcare research or DEIJ | 8 | 20 | 25 | 28 |
| Full Text Screening | In-depth discussion of facilitators, barriers, and recommendations to improve DEIJ in grantmaking for healthcare research | Full text not in English; lack of focus on healthcare research or DEIJ | 18 |  | 10 | 18 |
| Citation Searching of Accepted Articles | Meets all above inclusion criteria | Does not violate any exclusion criteria | 8 |  |  | 26 |
